# Identification of calcium-binding proteins associated with the human sperm plasma membrane

**DOI:** 10.1186/1477-7827-8-6

**Published:** 2010-01-15

**Authors:** Soren Naaby-Hansen, Alan Diekman, Jagathpala Shetty, Charles J Flickinger, Anne Westbrook, John C Herr

**Affiliations:** 1 Department of Clinical Immunology, Aalborg Sygehus, Århus University Hospital, Aalborg, Denmark; 2 Department of Biochemistry and Molecular Biology, University of Arkansas for Medical Sciences, Little Rock, Arkansas, USA; 3 Department of Cell Biology, University of Virginia, Charlottesville, VA, USA; 4 Telemedicine and Advanced Technology Research Center, Ft Detrick, MD, USA

## Abstract

**Background:**

The precise composition of the human sperm plasma membrane, the molecular interactions that define domain specific functions, and the regulation of membrane associated proteins during the capacitation process, still remain to be fully understood. Here, we investigated the repertoire of calcium-regulated proteins associated with the human sperm plasma membrane.

**Methods:**

Surface specific radioiodination was combined with two-dimensional gel electrophoresis, a 45Ca-overlay assay, computer assisted image analysis and mass spectrometry to identify calcium-binding proteins exposed on the human sperm surface.

**Results:**

Nine acidic 45Ca-binding sperm proteins were excised from stained preparative 2D gels and identified by mass spectrometry. Five of the calcium binding proteins; HSPA2 (HSP70-1), HSPA5 (Bip), HYOU1 (ORP150), serum amyloid P-component (SAP) and protein kinase C substrate 80K-H (80K-H) were found to be accessible to Iodo-Bead catalyzed 125I-labelling on the surface of intact human sperm. Agglutination and immunofluorescence analysis confirmed that SAP is situated on the plasma membrane of intact, motile sperm as well as permeabilized cells. Western blot analysis showed increased phosphorylation of human sperm 80K-H protein following in vitro capacitation. This is the first demonstration of the 80K-H protein in a mammalian sperm.

**Conclusion:**

The presence of SAP on the surface of mature sperm implies that SAP has a physiological role in reproduction, which is thought to be in the removal of spermatozoa from the female genital tract via phagocytosis. Since 80K-H is a Ca2+-sensor recently implicated in the regulation of both inositol 1,4,5-trisphosphate receptor and transient receptor potential (TRP) cation channel activities, its detection in sperm represents the first direct signaling link between PKC and store-operated calcium channels identified in human sperm.

## Background

The composition and regulation of the plasma membrane (PM) of mammalian sperm have been subjects of numerous studies, which have facilitated the identification and characterization of a variety of gamete surface molecules. The study of the sperm surface is complicated, however, by the organization of the plasma membrane into several distinctive domains, each with its own composition and function, by its complement of unique testis-specific proteins, which may be auto or iso-antigenic in males and females, and by the addition of secretory proteins originating in the male sex accessory glands. As a consequence, the precise composition of the sperm surface, the molecular interactions that define domain specific functions, and the changes induced during the capacitation process, still remain to be fully elucidated.

Among physiologically important sperm surface molecules, the plasma membrane receptor(s) that mediates zona pellucida (ZP)-binding has not been unequivocally identified [1,2], and the receptor-induced signaling cascade that culminates in acrosomal exocytosis remains to be fully elucidated. Calcium influx, however, is an absolute requirement for physiological induction of the acrosome reaction (AR) in all mammalian sperm [3]. ZP-binding generates a biphasic calcium response in sperm, which is currently thought to involve at least three separate, yet sequentially linked, Ca^2+ ^channels [2,4]. Activation of the putative ZP-receptor leads to a transient influx of calcium through T-type voltage-dependent calcium channels in the plasma membrane that are thought to be released from inactivation by the capacitation-induced hyperpolarization of the membrane potential [5]. This brief (< 500 ms) initial elevation of [Ca^2+^]_i _to micromolar levels activates the Ca^2+^-sensitive phospholipase PLCδ, causing the generation of diacylglycerol (DAG) and inositol 1,4,5-triphosphate (IP_3_), and consumption of the plasma membrane positioned substrate phosphatidylinositol biphosphate (PIP_2_) [4,6]. The increased production of IP_3 _leads to the emptying of IP_3_-receptor regulated intracellular Ca^2+^-stores situated in the acrosome [7,8] and in membrane bounded calreticulin containing vesicles localized to the post-acrosomal region of human sperm [9]. Similar to what happens in somatic cells, the depletion of Ca^2+ ^from internal stores is thought to activate store-operated channels (SOC) in the sperm plasma membrane causing a sustained elevation in [Ca^2+^]_i _[7,10]. Increases in calcium, cAMP and small G protein activities act together to set in motion the SNARE machinery (soluble N-ethylmaleimide-sensitive factor attachment protein receptor), which is required for the fusion between the outer acrosomal membrane and the overlying plasma membrane [4].

Thus, one approach to understanding changes in the gametes that take place before and during fertilization is to study the cellular constituents of the calcium signaling pathways and their functions in sperm. In the present study we have combined a ^45^Ca-overlay assay with vectorial radiolabelling and mass spectrometry analysis, to identify calcium-binding proteins situated on the surface of freshly ejaculated human sperm. Nine calcium-binding 2D gel protein spots were detected on Coomassie stained preparative gels by computer-aided image analysis and were identified by mass spectrometry: CABYR, calreticulin, tubulin, calmodulin, HYOU1, HSPA5, HSPA2, serum amyloid P-component (SAP), and 80K-H. The latter five were found to be accessible to Iodo-Bead catalyzed ^125^I-labelling of intact, motile sperm and therefore were considered to be on the sperm surface. Members of four different heat shock protein families, including HYOU1, HSPA2 and HSPA5, have previously been detected on the surface of swim-up harvested human sperm [11], and the heat shock proteins have been amply studied in other contexts, so attention was focused on SAP and 80K-H. SAP is present in human testis and on the surface of mature sperm from healthy young men, suggesting that it has a physiological role in reproduction [12]. On the other hand, this is the first detection of the Ca^2+ ^sensor 80K-H in mammalian sperm, where it may be a link between PKC and store-operated calcium channels.

## Methods

### Preparation and labelling of human sperm

Semen specimens were obtained from normal, healthy young men by masturbation. Only ejaculates with normal semen parameters (WHO criteria) were used in this study. Individual semen samples from five selected donors were allowed to liquify at room temperature (normally for 1 h, range 0.5 to 3 h) before the motile sperm were separated from seminal plasma, immature germ cells and non-sperm cells by the swim-up method [13]. All samples were obtained under informed consent using forms approved by the University of Virginia Human Investigation Committee. In some experiments the harvest was concentrated by density gradient centrifugation employing a discontinuous 55%/80% Percoll gradient, and was then resuspended in human tubal fluid (Irvine, Santa Ana, CA) containing human serum albumin (30 mg/ml) and 100 μM progesterone. Capacitation was achieved by incubating the samples at 37°C in 5% CO_2_. Samples were removed at various time points and isolated by centrifugation. Control samples of Percoll concentrated swim-up harvested sperm were removed and snap frozen prior to *in vitro *capacitation. Iodo-Bead (Pierce) catalyzed ^125^I-labelling of Percoll concentrated swim-up harvested sperm was performed as previously described [14].

### Electrophoresis and analysis of spermatozoa proteins

Purified sperm were solubilized in a lysis buffer containing: 2% (v/v) NP-40; 8.8 M urea; 100 mM DTT; 2% (v/v) ampholines pH 3.5-10; and the protease inhibitors 2 mM PMSF, 3 mg/ml TLCK, 1.46 mM pepstatin A and 2.1 mM leupeptin. 5 × 10^8 ^cells per ml were solubilized by constant shaking at 4°C for 60 min. Insoluble material was removed by centrifugation at 10,000 × g for 2 min, and the supernatant used for first dimension electrophoresis. Protein concentrations were determined by using the bicinchoninic acid method (Pierce, Rockford, IL), employing bovine serum albumin as the standard.

Analytical two-dimensional gel electrophoresis was performed as previously described [14]. Preparative two-dimensional gel electrophoresis in large format gels (23 × 23 cm) was performed in an 'Investigator 2-D Electrophoresis System' (Genomic Solution, UK), employing the following ampholine (Pharmacia) composition: 20% pH 5-7, 20% pH 7-9 and 60% pH 3.5-10.

Computerized pattern analysis and densitometry of autoradiograms and stained gels and membranes were performed employing the 2D Analyzer software (BioImage 2000). All radiolabeling experiments were replicated at least four times. Protein targets that had been identified by image comparison were carefully excised from Coomassie stained preparative 2-D gels, subjected to in-gel trypsinization, and analysed by LC-electrospray-tandem mass spectrometry [15].

Electrotransfer to PVDF membranes (0.2 μm pore size, Pierce) was carried out as previously described [16] using the transfer buffer composition of Matsudaira [17] (10 mM 3- [cyclohexylamino]-1-propanesulfonic acid, 10% methanol, pH 11). PVDF immobilized proteins were visualized by staining the membrane in a solution containing 0.1% Coomassie R250, 40% methanol and 0.1% acetic acid for one minute, followed by destaining in a solution of 10% acetic acid and 50% methanol for 3 × 3 minutes. The center of each selected Coomassie stained spot was carefully cut from the PVDF membrane and microsequenced by Edman degradation.

Calcium binding proteins were detected using a ^45^Ca overlay assay modified from that described by Maruyama and colleagues [18]. The use of PVDF and the employment of phospho-imaging detection increased the signal to noise ratio compared to that achieved with NC-paper and X-ray films [9]. Some of the PVDF membranes were subsequently stained with Coomassie blue to localize the calcium binding proteins within the global pattern of 2DE separated protein species, while other membranes were used for western blot analysis.

A 1:2500 dilution of the anti-phosphotyrosine monoclonal antibody RC-20 (Transduction Laboratories) was used in western blots, while rabbit antiserum against SAP was used in a 1:2000 dilution. In some experiments secondary antibodies were employed alone as a control. Immunostaining was preceded by gold colloid staining of the NC-membrane of other blots to localize individual antigens within the global pattern of sperm proteins.

### Immunofluorescence staining of human spermatozoa

For immunofluorescence studies of non-permeabilized motile cells, fresh human spermatozoa were harvested over a discontinuous 55%/80% Percoll gradient and subsequently washed three times with Ham's F-10 medium. The sperm were counted using a hemocytometer and diluted to a concentration of 1 × 10^6 ^sperm/ml, and incubated with a rabbit antiserum against human SAP for 2 h (1:400 dilution), while the secondary antibody, a goat anti-rabbit IgG TRITC conjugate (Jackson ImmunoResearch), was applied at a 1:200 dilution for 1 h at 37°C. DAPI II was utilized to stain the sperm DNA.

For immunofluorescent staining of permeabilized sperm, the Percoll harvested, washed spermatozoa were air dried and permeabilized with methanol for 10 minutes. The sperm were treated with 10% normal goat serum for 1 h at 22°C and incubated with either rabbit antiserum against human SAP (dilution 1:100) followed by the secondary antibody, a goat anti-rabbit IgG FITC conjugate (Jackson ImmunoResearch), or the secondary antibody alone for 1 h at 22°C. The slides were washed and mounted with Slow-Fade antifade reagent (Molecular Probes, Eugene, OR) containing DAPI. Images were captured using a Zeiss Axioplan2 microscope (Carl Zeiss Inc., Thornwood, NY).

### Sperm agglutination assay

The standard slide agglutination assay was performed as previously described [19]. Human semen samples were liquefied at room temperature. One part of semen diluted to 40 × 10^6 ^sperm/ml in Ham's F-10 medium was gently mixed with one part of anti-SAP polyclonal antiserum diluted 1:5 in Ham's F-10 medium. Ham's F-10 alone was included as a negative control. A sperm agglutinating monoclonal antibody was utilized as a positive control [19]. Twenty microliters of each mixture were placed on a hemocytometer with a coverslip. Sperm agglutination was observed and recorded with differential interference contrast microscopy (DIC) using a Zeiss Axioplan microscope (Carl Zeiss, Inc., Thornwood, NY) equipped with a digital camera.

## Results

**Figure 1:**
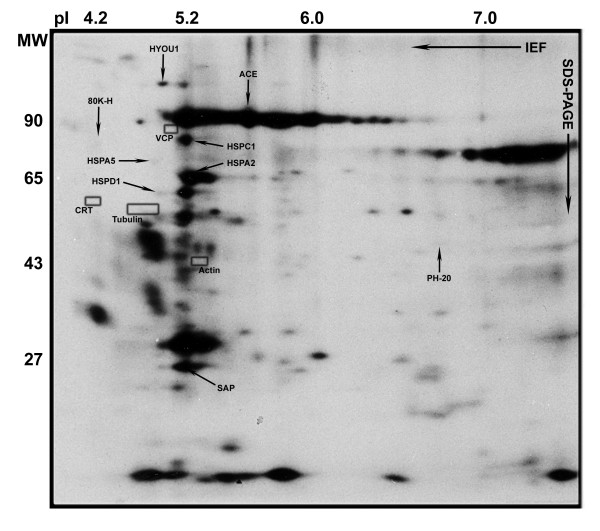
**2D autoradiogram of radioiodinated acidic and neutral human sperm surface proteins**. The positions of 80K-H and SAP are indicated by arrows, as well as those of previously reported surface proteins; oxygen regulated protein 150 (HYOU1), angiotensin converting enzyme (ACE), HSP86 (HSPC1), Bip (HSPA5), sperm-specific surface hyaluronidase (PH-20) and HSP70-1 (HSPA2). The positions of four intracellular proteins; valosine containing protein (VCP), calreticulin (CRT), tubulin and actin are indicated by rectangular boxes. Additional non-labelled tubulin isoforms were detected by mass spectrometry to the right of the boxed area. See also Figure 3A and Additional file 1 - Supplementary Figure 1.

Progressively motile human spermatozoa were harvested by the swim-up method, and surface-accessible phenols were labelled by Iodo-Bead catalyzed, electrophilic addition of cationic ^125^I [14]. After being removed from the Iodo-Beads the cells were subjected to Percoll density gradient centrifugation and washed three times with Ham's F-10 medium, to ensure that only proteins tightly bound to the plasma membrane were included in the study. Slightly more than one hundred radiolabelled protein spots with MW between 5 and 200 kDa were detected by autoradiography after IEF/PAGE (3.0 < pI < 8.5) separation (Figure 1). The cytosolic protein valosin-containing protein (VCP) and calreticulin (CRT), which localize to intracellular vesicles in the neck of human sperm, and the cytoskeletal proteins tubulin and actin were not radioiodinated by the vectorial labelling procedure (indicated by dark rectangles in Figure 1). Conversely, angiotensin converting enzyme (ACE), previously demonstrated to be attached to the human sperm plasma membrane [20], the sperm specific GPI-anchored surface hyaluronidase PH-20 [21], as well as known components of both somatic and gamete cell surfaces, including several members of the heat shock protein (HSP) family [11,22-25], were all consistently labelled with radioiodine (denoted by black arrows in Figure 1), indicating that the employed procedure labelled surface exposed species.

**Figure 2:**
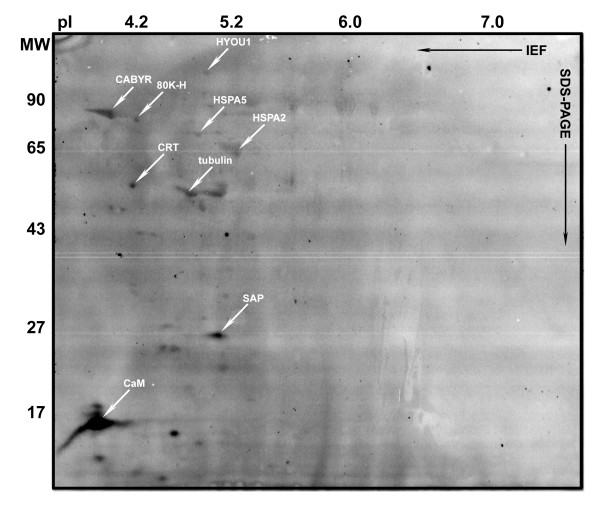
**2D autoradiogram demonstrating ^45^Ca-binding human sperm proteins**. The nine identified calcium binding proteins are indicated by oblique arrows.

**Figure 3:**
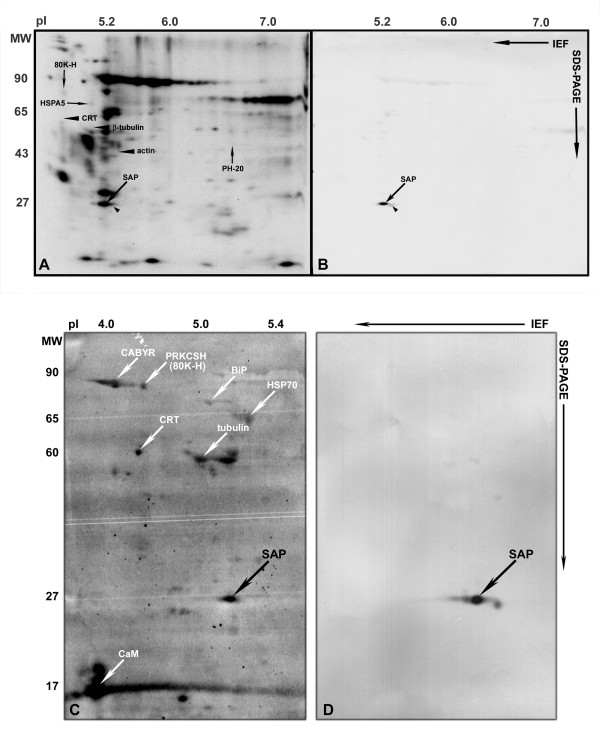
**SAP is a ^45 ^Ca-binding protein exposed on the surface of human sperm**. Immuno-staining of PVDF membranes used to detect radioiodinated surface proteins (A) and ^45^Ca-binding proteins (C) by autoradiography confirmed that the 26.5 kDa calcium binding surface protein with a pI of 5.2 (oblique downward arrows) is SAP (B & D).

Calcium binding proteins (CBPs) of human sperm were identified by the modified ^45^Ca-overlay procedure [9]. More than a dozen proteins with molecular weights between 5 and 120 kDa, and isoelectric points ranging from 3 to 6, were found to bind ^45^Ca (Figures 2 and 3C). Nine calcium-binding protein spots were carefully excised from complementary stained gels and PVDF membranes, and identified by mass spectrometry and/or Edman degradation analysis. The nine calcium-binding proteins thus identified in detergent/urea extracts of human sperm are given in Figure 2. The protein with the highest relative ^45^Ca-binding capacity was identified as calmodulin (CaM), the major calcium-binding component of the mammalian sperm cytosol.

Computer comparison of 2D images of calcium-binding spots with images of proteins vectorially labelled with radioiodine and images of 2D gels where the proteins had been visualized by Coomassie or silver staining, allowed identification of calcium binding proteins exposed on the sperm surface. Five calcium binding proteins; HYOU1, HSPA5, HSPA2, SAP and 80K-H were found to be accessible to Iodo-Bead catalyzed radiolabelling. The three calcium binding HSP70 chaperones HYOU1, HSPA5 and HSPA2 were recently shown to be accessible to biotin labelling on the surface of motile human sperm [11].

The 80 kDa calcium-binding surface protein migrating at a pI of 4 was identified as 80K-H (Figures 1, 2 and 3), a phosphoprotein containing two calcium-binding helix-loop-helix structures. Radiolabelling of 80K-H was highly reproducible, although the amount of iodine-isotopes incorporated into the protein was sparse (see Figures 1 and 3A, and Additional file 1 - Supplementary Figure 1). 80K-H contains several potential threonine and tyrosine phosphorylation sites, and increased phosphorylation of the protein was observed following *in vitro *capacitation of human sperm (see Additional file 2 - Supplementary Figure 2). Efficient induction of *in vitro *capacitation was confirmed by the significant increase in tyrosine phosphorylation of CABYR, fibrous sheath protein 95 (FSP95, AKAP3) and valosin-containing protein/p97 (VCP) [15,26]. The capacitation-induced phosphorylation of 80K-H did not alter the protein's ^45^Ca binding capacity (see Additional file 3 - Supplementary Figure 3).

Densitometry of the autoradiograms showed that the abundant surface protein SAP (MW 26.5 kDa, pI 5.2) accounts for more than six per cent of the ^45^Ca binding capacity in the acidic and neutral pH range of the human sperm proteome, thus identifying SAP as the surface labelled constituent that binds relatively most ^45^Ca in the overlay assay (Figures 1, 2 and 3). Immuno-staining of the PVDF membranes used for ^45^Ca and ^125^I autoradiography confirmed that the 26 kDa surface labelled calcium-binding protein was SAP (Figure 3). In addition to the major ^45^Ca-binding form, a slightly more basic and at least one slightly more acidic form of the SAP antigen was revealed by the Western blot analysis (see Figure 3D and Additional file 4 - Supplementary Figure 4). SAP is a glycoprotein with a single N-glycosylation site, at Asn 32, which in the native protein contains a typical complex biantennary oligosaccharide chain [27]. Structural variants of SAP which lack one or both terminal sialic acid residues have been found in human plasma and urine [28], suggesting that the charge variants of human sperm SAP might be due to micro heterogeneity of the glycan structure.

**Figure 4:**
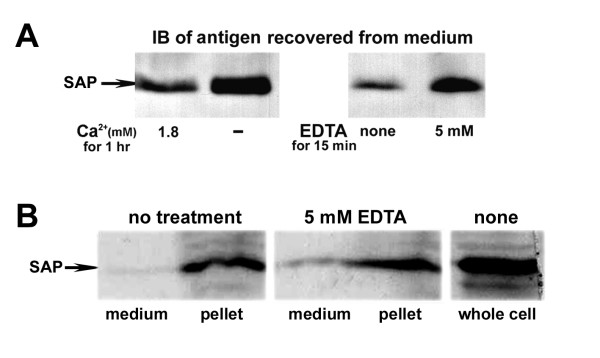
**The majority of SAP molecules bind to the human sperm surface in a calcium-independent fashion**. SAP is slowly released from the surface of washed, swim-up harvested human sperm when incubated in a Ca^2+^-free DMEM medium (A, left panel). The addition of 5 mM EDTA to the medium induced a similar strong discharge of surface bound SAP within minutes (A, right panel). However, most SAP antigens remained attached to the sperm, despite the removal of calcium from the medium (B).

Western blot analyses showed that the concentration of SAP in the medium from sperm incubated in the absence of calcium for 1 hr at 37°C was several fold higher than that released from sperm incubated in the presence of 1.8 mM CaCl_2 _for a similar period (Figure 4A, left). Moreover, the addition of 5 mM EDTA to fresh, motile human sperm induced a similar release of surface attached SAP within minutes (Figure 4A, right). However, less than 10% of the gamete's total SAP content was released by EDTA treatment (Figure 4B), indicating that the majority of SAP molecules are associated with the human sperm in a calcium-independent manner.

**Figure 5:**
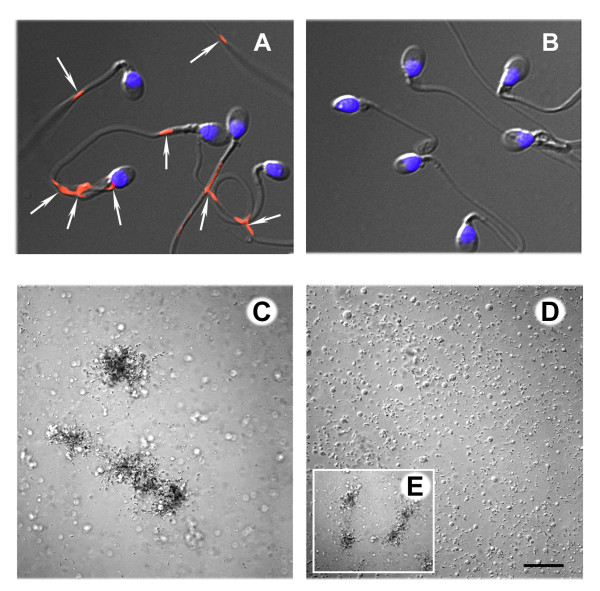
**Demonstration of SAP on the surface of non-permeabilized, motile sperm**. A: IF image demonstrating patches of SAP over the neck and tail regions of intact, motile human sperm (arrows). The nuclear DNA is stained blue with the DNA intercalating dye DAPI II. B: Secondary antibody alone control. C: Sperm agglutination floccules visualized by differential interface contrast microscopy. Antiserum against SAP agglutinated human sperm in a loose, tangled binding pattern, i.e. tail-to-tail, head-to-tail, and head to head. Round cells did not appear to be incorporated into the agglutination floccules. D: No antibody control without agglutination. Scale bar = 100 μm. E: A positive control with monoclonal antibody against CD52 induced strong agglutination.

Immunofluorescence (IF) microscopy confirmed the presence of SAP on the surface of intact, viable human sperm (Figure 5). SAP was localized to the membrane overlying the neck, midpiece and tail regions of fresh, motile sperm (Figure 5A). The patches of SAP staining were confined to the proximal section of the principal piece in the majority of cases. IF staining of permeabilized fixed sperm revealed intracellular SAP antigen in the neck region of some human sperm (see Additional file 5 - Supplementary Figure 5).

Antiserum against SAP induced mixed agglutination of swim-up harvested human sperm in the standard slide agglutination assay (Figure 5C), consistent with the broad distribution of the antigen observed by IF. The mixed agglutination pattern obtained with highly motile cells implies that SAP is tightly bound to the plasma membrane overlying both the neck and tail regions of human sperm.

## Discussion

### Calcium-binding proteins detected by ^45^Ca-binding assay

Five of the nine human sperm ^45^Ca-binding proteins identified in this study contain at least one calcium binding helix-loop-helix structure (EF-hand). This suggests that the EF-hand is either resistant to the denaturing effect of SDS to which the proteins are exposed to during the second dimension electrophoresis or that the domain is readily refolded during the subsequent mild electrotransfer, washing and blocking procedures. Both tubulin and HSP70 chaperones, which lack an EF-hand domain, have previously been shown to bind calcium [15,29-32], confirming the specificity of the ^45^Ca-overlay procedure employed in the present study.

Five of the calcium-binding proteins were found to be accessible for radioiodination on the surface of ejaculated human sperm: HYOU1, HSPA5, HSPA2, SAP, and 80K-H. The three heat shock protein 70 family-members HYOU1, HSPA5 and HSPA2 have previously been demonstrated on the surface of both the male [11,24] and the female gamete [23]. Indeed, HSP70 antigens have been localized over the entire human sperm surface by immunofluorescence analysis [33].

### Serum amyloid P-component (SAP) in association with spermatozoa

SAP has been localized to the human sperm surface by vectorial labelling, immunohistochemistry, and flow cytometry analysis [15,12]. The presence of SAP in the sperm-free seminal fluid from a vasectomized man suggested that SAP associates with the sperm membrane after the epididymal contents mix with secretions from the accessory glands [15]. However, SAP encoding mRNA was recently isolated from human testis, seminal vesicle and epididymis, indicating local synthesis of SAP in all three organs [12]. Moreover, SAP antigens were localized to the seminiferous tubules containing late spermatids by immunohistochemistry [12], and epididymal sperm and epithelial cells were also strongly positive for SAP, suggesting that at least some SAP antigen associates with the sperm membrane during the later stages of spermatogenesis and/or the epididymal maturation process [12].

The results from the experiments in which sperm were treated with EDTA indicate that most SAP molecules are attached to the human sperm membrane in a calcium-independent manner (Figure 4). SAP can bind to glycosaminoglycans and amyloid proteins in a calcium-independent manner [34,35], and it associates with microbial polysaccharides and extracellular matrix components through carbohydrate determinants, including heparin and 6-phosphorylated mannose. However, whether SAP's membrane attachment involves carbohydrate structures on the sperm surface, or occurs through interaction with other molecules situated in the outer leaflet of the sperm plasma membrane (e.g. phosphatidylethanolamine), remains to be determined.

SAP can activate the classical complement pathway via interaction with C1q [36], and complement components on the head of acrosome reacted sperm have been suggested to facilitate sperm-egg binding via complement receptors on the egg surface [37]. However, SAP is an unlikely participant in such interactions as it mainly localizes to the neck and tail regions of intact, washed human sperm, and IF staining of permeabilized sperm failed to detect SAP antigens in the acrosome compartment (see Additional file 5 - Supplementary Figure 5).

Recent studies suggest that SAP can act as an opsonin [38-41], facilitating the uptake of apoptotic cells by direct interaction with the Fcγ receptors on macrophages [42,43]. Binding of SAP and other members of the innate immune system to the asymmetric pattern of phospholipids found on apoptotic cells is also thought to have important immuno-modulatory effects on the ingesting phagocytes, triggering them to release anti-inflammatory cytokines rather than to produce inflammatory cytokines, thereby collaborating T-cell suppression and the maintenance of tolerance [44-46].

SAP binding and stabilization of cellular debris and soluble immune complexes thus appear to facilitate their subsequent clearance by phagocytes [47,48]. In addition, SAP binds DNA and chromatin with high affinity and avidity [49], and it has been proposed that SAP's chaperone-like binding and stabilization of nuclear macromolecule antigens protect them from proteolysis and prevent subsequent spread of immunogenic degradation products [50]. Since mammalian spermatozoa are removed from the female genital tract via phagocytosis, mainly mediated by invading leukocytes and macrophages [51-53], these observations suggest the speculation that SAP participates in a molecular mechanism that facilitates the disposal of sperm remnants from the female genital tract, while at the same time ensuring that repetitive clearance of isoantigenic sperm and their cargo of super-coiled DNA by professional phagocytes occurs without triggering severe inflammatory or antinuclear autoimmune responses.

### 80K-H protein

Several studies suggest that store-operated calcium channels in mammalian sperm belong to the transient receptor potential (TRP)-family of cation channels, whose members are closely related to the TRP gene expressed in Drosophilia photoreceptors [54]. Five members of the TRP channel family have been detected in mammalian sperm [6,10,55], of which 4 localize to the head of the human sperm [4]. More important, maitotoxin, which induces Ca^2+^-uptake through its action on TRP channels, is the most potent inducer of the acrosome reaction in mouse sperm aside from ZP [56]. TRPC2 has been proposed to participate in the sustained Ca^2+ ^influx triggered by ZP3 in mouse sperm [10], although it appears to be a pseudogene in humans [57,58]. TRPC channels can form heteromultimers [59], and it is likely that the store-operated Ca^2+ ^entry pathway in sperm involves several family members, which can at least partly substitute for each others, as TRPC2 null mice are fertile [60,61].

Several TRP channel regulating molecules have been identified, including STIM [62], junctate [63], PIP_2 _[64], enkurin [65], and 80K-H [66]. While enkurin and junctate previously have been detected in mouse sperm [63,65], this is the first demonstration of the 80K-H protein in a mammalian sperm.

80K-H is a multifunctional Ca^2+^-sensor originally identified as a substrate for PKC [67]. 80K-H has been associated with the regulation of intracellular signaling downstream of both the fibroblast growth factor receptor [68,69] and the advanced glycosylation end products receptor [70], and it participates in the regulation of protein translocation [71]. 80K-H interacts with PKCζ and munc18c to induce glucose transporter 4 translocation to the plasma membrane [72]. A recent study suggests that 80K-H can regulate IP_3_-induced calcium release by interacting with the cytoplasmic tail of IP_3_-receptors [73]. Finally, 80K-H has been shown to interact with and regulate the activity of the epithelial TRP channel V5 (TRPV5) [66]. The plasma membrane density and activity of TRPV5 channels appear to be regulated via changes in their extracellular glycosylation status [74]. Processing of specific N-linked carbohydrate sidechains from the ectodomain of TRPV5 channels is thought to entrap them in the plasma membrane, resulting in increased Ca^2+ ^influx [75,76]. This is noteworthy, as 80K-H acts as the regulatory subunit of α-glucosidase II, an N-linked glycan-processing enzyme [77,78].

Several studies have indicated a major role for PKC in the upregulation of cytosolic calcium levels prior to the AR in human sperm [79-82], and it has been suggested that PKC participates in the opening of store-operated calcium channels in the sperm plasma membrane [83,84]. However the molecular mechanism by which PKC controls capacitative calcium entry has remained elusive.

Identification of the PKC substrate 80K-H in the human sperm proteome thus denotes the first putative effector molecule which directly links PKC to both the regulation of intracellular calcium stores and the opening of store-operated calcium channels in the sperm plasma membrane. The presence and phospho-regulation of sperm 80K-H support the notion that store-operated calcium channels in human sperm belong to the TRP channel superfamily, and suggest that PKC might increase and sustain Ca^2+^-influx prior to the acrosome reaction through 80K-H-mediated upregulation and stabilization of active TRP channels in the plasma membrane.

### Calcium binding proteins

In this study a combination of surface protein labeling, two-dimensional gel electrophoresis, a ^45^Ca-overlay assay, and mass spectrometry led to the identification of five calcium binding proteins exposed on the surface of the human sperm plasma membrane. Although functionally interesting, none of the identified proteins possess a membrane spanning hydrophobic domain. Hydrophobic membrane proteins are known to be underrepresented on 2D gels [85], which may explain why no integral calcium-binding membrane proteins (e.g. TRP-family members) were detected by this experimental approach. One way this restriction can be circumvented is to use unidirectional gel electrophoresis (SDS-PAGE) separation of affinity purified membrane proteins [25] in future ^45^Ca overlay studies of human sperm.

## Competing interests

The authors declare that they have no competing interests.

## Authors' contributions

SNH performed design, analysis and reporting of study, acquisition of the data presented in Figures 1, 2, 3, 4A, and the Additional files - supplementary material section. AD performed acquisition of the data presented in Figures 5C-E. JS performed acquisition of the data presented in Figure 4B and Additional file 5 - Supplementary Figure 5. CJF performed revision of the manuscript. VAW performed acquisition of the data presented in Figures 5A-B. JCH organized the program and grant funding, and performed revision of the manuscript. All authors read and approved the final manuscript.

## Supplementary Material

Additional file 1 **Supplementary Figure 1**. Enlarged area of 2D autoradiograms demonstrating weak but reproducible radiolabelling of the 80K-H protein (PRKCSH) and Bip on the surface of human sperm. Click here for file

Additional file 2 **Supplementary Figure 2**. WB detection of tyrosine phosphorylated proteins in fresh human sperm (left) and in sperm subjected to an *in vitro *capacitation medium for 6 hrs (right). Click here for file

Additional file 3 **Supplementary Figure 3**. Enlarged area of 2D autoradiograms demonstrating ^45^Ca-binding to the 80K-H protein in extracts from fresh (left) and capacitated human sperm (right). *In vitro *capacitation did not alter the ^45^Ca-binding capacity of the protein significantly. Click here for file

Additional file 4 **Supplementary Figure 4**. Neutral and acidic human sperm proteins were separated by 2D gel electrophoresis (IEF/PAGE), and visualized by silver staining (A) or by colloidal gold staining following their transfer to a NC-membrane (B). Subsequent immuno-staining of the immobilized proteins with an antibody against SAP facilitated the identification of the different isoforms on silver stained gels. Click here for file

Additional file 5 **Supplementary Figure 5**. Immunofluorescent detection of SAP in permeabilized human sperm. A: A punctuate immunofluorescence (green) was noted on the neck region of some sperm demonstrating the retention of SAP in methanol permeabilized sperm. The DAPI stained nuclear DNA is stained blue. B: Secondary antibody alone control. Click here for file
