# Supplementary figures and images for: Identification of calcium-binding proteins associated with the human sperm plasma membrane
Source: Reprod Biol Endocrinol. 2010 Jan 15;8:6. doi: 10.1186/1477-7827-8-6 (PMC2822784; doi:10.1186/1477-7827-8-6)

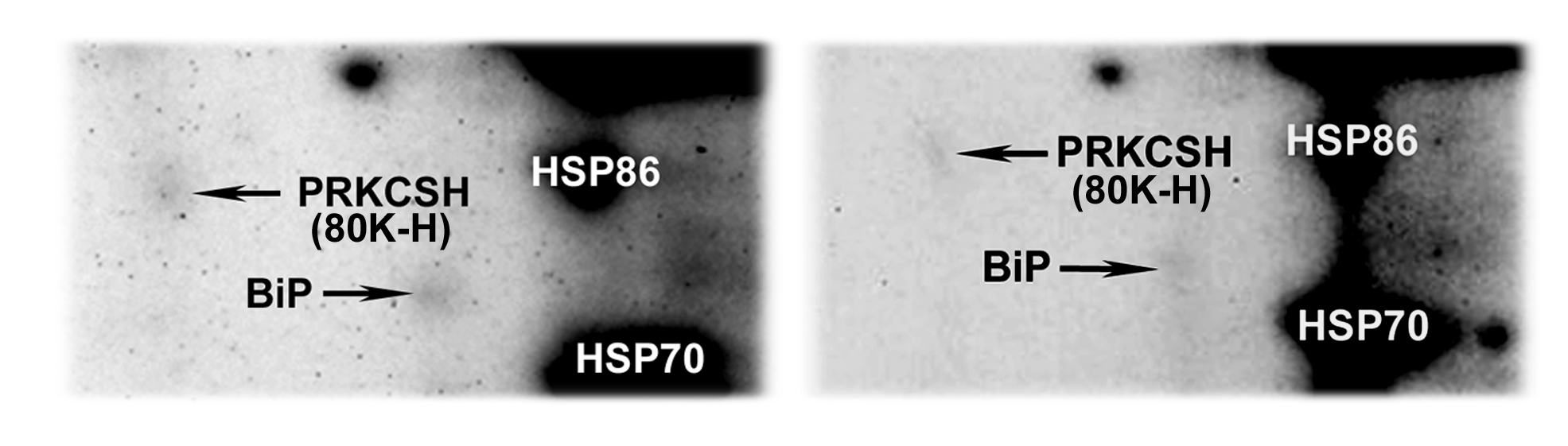

Supplement: Additional file 1 — Supplementary Figure 1. Enlarged area of 2D autoradiograms demonstrating weak but reproducible radiolabelling of the 80K-H protein (PRKCSH) and Bip on the surface of human sperm. [file 1477-7827-8-6-S1.TIFF]

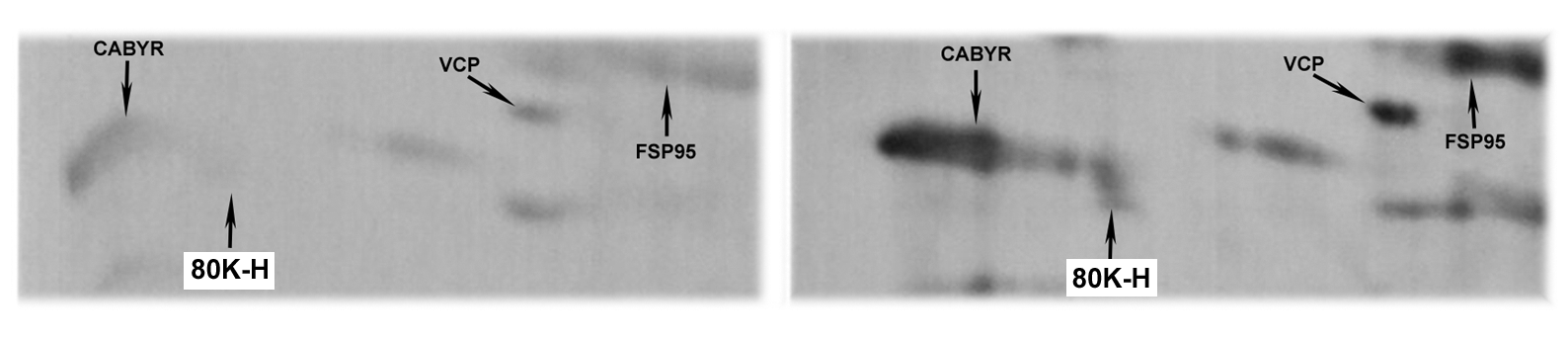

Supplement: Additional file 2 — Supplementary Figure 2. WB detection of tyrosine phosphorylated proteins in fresh human sperm (left) and in sperm subjected to an in vitro capacitation medium for 6 hrs (right). [file 1477-7827-8-6-S2.TIFF]

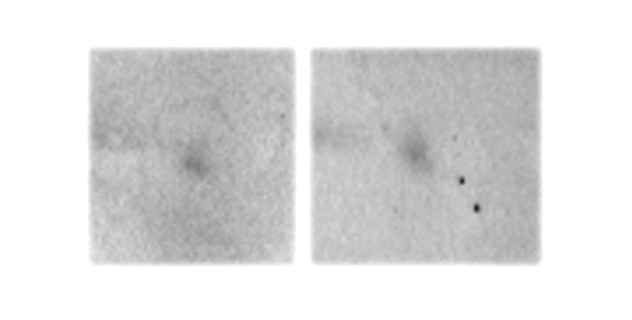

Supplement: Additional file 3 — Supplementary Figure 3. Enlarged area of 2D autoradiograms demonstrating 45Ca-binding to the 80K-H protein in extracts from fresh (left) and capacitated human sperm (right). In vitro capacitation did not alter the 45Ca-binding capacity of the protein significantly. [file 1477-7827-8-6-S3.TIFF]

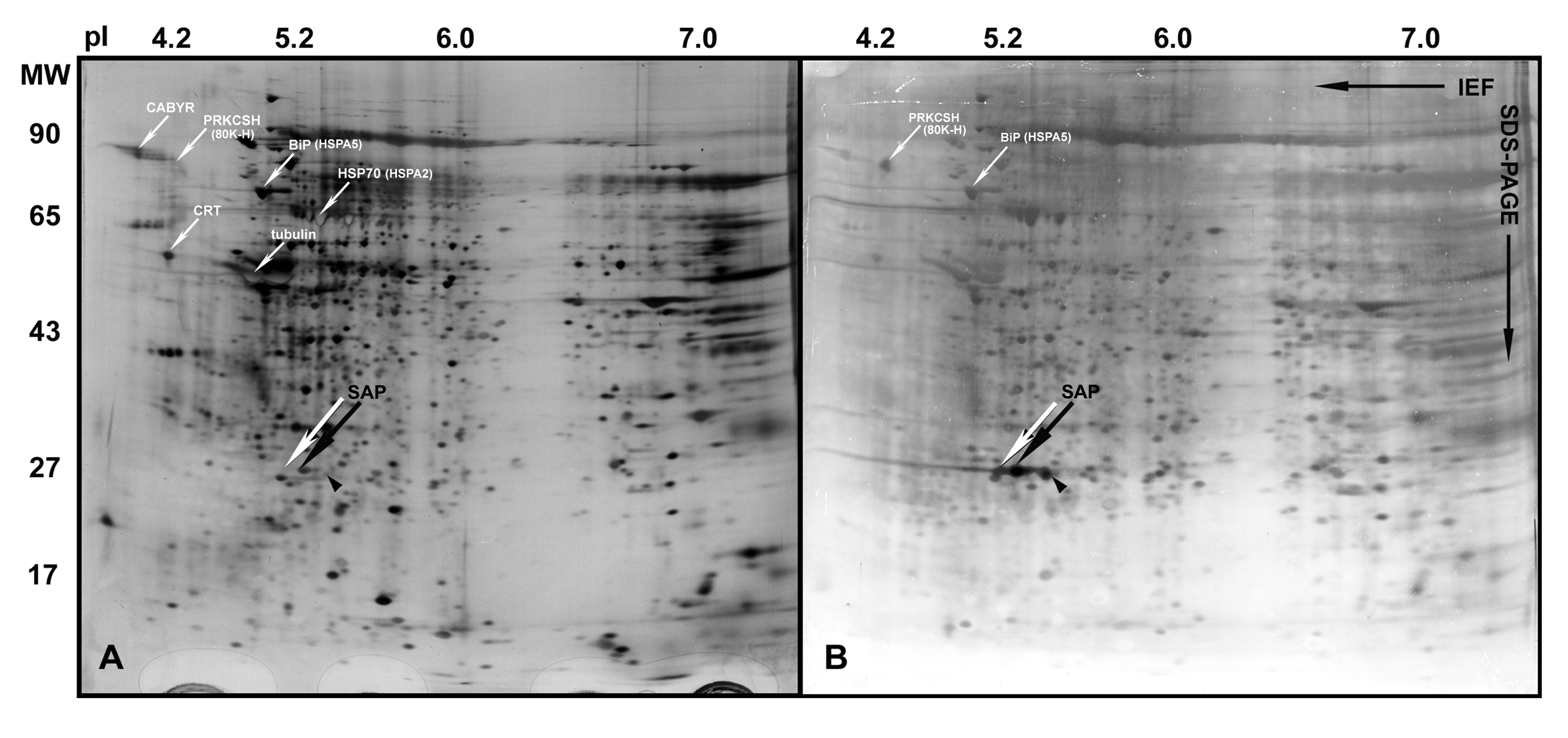

Supplement: Additional file 4 — Supplementary Figure 4. Neutral and acidic human sperm proteins were separated by 2D gel electrophoresis (IEF/PAGE), and visualized by silver staining (A) or by colloidal gold staining following their transfer to a NC-membrane (B). Subsequent immuno-staining of the immobilized proteins with an antibody against SAP facilitated the identification of the different isoforms on silver stained gels. [file 1477-7827-8-6-S4.TIFF]

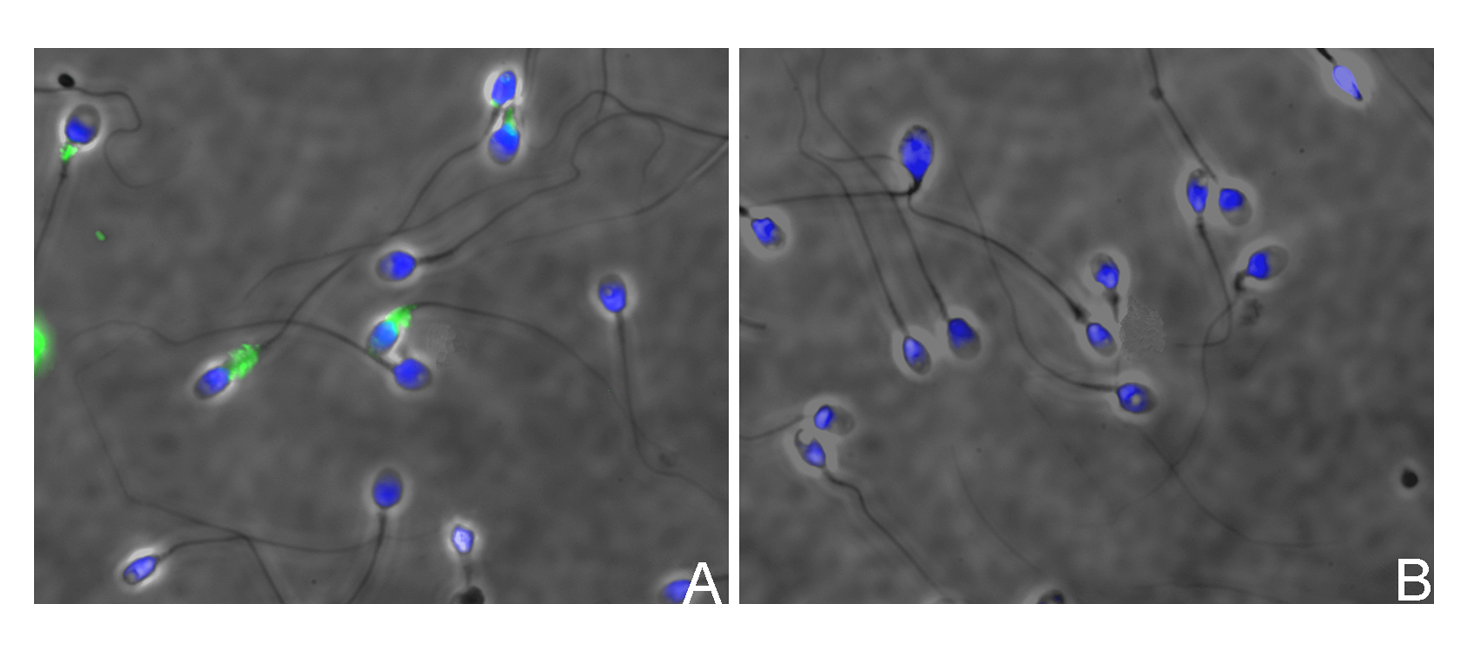

Supplement: Additional file 5 — Supplementary Figure 5. Immunofluorescent detection of SAP in permeabilized human sperm. A: A punctuate immunofluorescence (green) was noted on the neck region of some sperm demonstrating the retention of SAP in methanol permeabilized sperm. The DAPI stained nuclear DNA is stained blue. B: Secondary antibody alone control. [file 1477-7827-8-6-S5.TIFF]
